# Minds Under Siege: Cognitive Signatures of Poverty and Trauma in Refugee and Non‐Refugee Adolescents

**DOI:** 10.1111/cdev.13320

**Published:** 2019-10-24

**Authors:** Alexandra Chen, Catherine Panter‐Brick, Kristin Hadfield, Rana Dajani, Amar Hamoudi, Margaret Sheridan

**Affiliations:** ^1^ Harvard University; ^2^ Yale University; ^3^ Queen Mary University of London; ^4^ Hashemite University; ^5^ University of Wisconsin–Madison; ^6^ University of North Carolina at Chapel Hill

## Abstract

The impacts of war and displacement on executive function (EF)—what we might call the cognitive signatures of *minds under siege*—are little known. We surveyed a gender‐balanced sample of 12‐ to 18‐year‐old Syrian refugees (*n* = 240) and Jordanian non‐refugees (*n* = 210) living in Jordan. We examined the relative contributions of poverty, trauma exposure, posttraumatic stress, and insecurity to variance in inhibitory control and working memory. We observed associations between poverty and WM, suggesting that, even in populations exposed to substantial violence and fear, poverty is a specific pathway to WM deficit. We did not, however, find associations between EFs and exposures to trauma. Careful distinction between childhood adversities may illuminate which neurocognitive pathways matter for measures of cognitive function.

In this study, we seek to understand what might be called the cognitive signatures of *minds under siege*—how young minds are affected by exposures to war and protracted displacement. We examine executive functions (EF), a set of higher order cognitive skills necessary for abstract thinking, decision making, and executing complex plans (Diamond, 2013; Gruszka, Szymura, & Matthews, 2010), known to associate with improved psychosocial health (McTeague, Goodkind, & Etkin, 2016), academic performance (Blair & Razza, 2007; Patrick, Blair, & Maggs, 2008), and success on the labor market (Mercy Corps, 2014). Two critical component processes of EF are inhibitory control (IC) and working memory (WM; Miyake & Friedman, 2012), which we measured in Syrian refugee and Jordanian non‐refugee adolescents in northern Jordan. Seventy‐one million children are displaced worldwide (UNHCR, 2019), and a whole generation of children and adolescents from Syria are at risk of compromised psychosocial well‐being (Save The Children, 2017). Researchers have emphasized that the Syrian crisis and other prolonged conflicts present urgent challenges for survival, health, and development pathways (Verma & Petersen, 2018). To better understand these pathways and the influence of war and displacement on EF, we examined the extent to which different indicators of adversity—poverty, war‐related trauma, posttraumatic stress, and insecurity—predicted EF in refugees and non‐refugees.

In the United States, poverty is robustly associated with EF deficits in childhood and adolescence (Daneri, Blair, Kuhn, & FLP Key Investigators, 2018; Haft & Hoeft, 2017; Wolf & Suntheimer, 2019). Recent work, however, shows that exposures to poverty and violence have distinct impacts on IC and WM (Lambert, King, Monahan, & McLaughlin, 2016; Sheridan, Peverill, Finn, & McLaughlin, 2017). Exposures to violence at home and in the community do not predict WM or IC task performance when controlling for poverty (Sheridan et al., 2017). These findings have been interpreted as distinguishing between the impact of adversities involving threat (“harm or threat of harm”) and those involving deprivation (“absence of expected inputs from the environment”), the latter being associated with low socioeconomic status (McLaughlin & Sheridan, 2016). To date, it is unknown whether exposures to violence and poverty have distinct impacts on EF in conflict‐affected children and adolescents, or whether the association between poverty and EF identified in other populations will hold true in this population.

In lower‐ and‐middle income countries (LMICs), few studies have been able to distinguish the cognitive signatures of poverty and violence, or for that matter, the cognitive signatures of posttraumatic stress and other psychosocial stressors. Adolescent and child refugees experience a host of adversities, including pervasive poverty, exposure to war‐related violence, and feelings of insecurity with regard to their safety and future opportunities (Verma & Petersen, 2018). These exposures are associated with mental health problems and difficulties in school (Dajani, Hadfield, van Uum, Greff, & Panter‐Brick, 2018; Miller‐Graff & Cummings, 2017; Wong, Schweitzer, & Khawaja, 2018). Few studies of conflict‐affected youth have measured performance on cognitive tasks, with the notable exception of work with Palestinian adolescents linking Intifada participation with attention and memory difficulties (Qouta, Punamäki, & Sarraj, 1995). For Syrian refugees, specifically, it is not known whether posttraumatic stress disorder (PTSD) or levels of insecurity are independently associated with cognitive performance, over and above exposure to traumatic events and poverty. This is important, as PTSD‐prevalence rates reported for child and adolescent refugees in the Middle East are high (41%–87%; Reed, Fazel, Jones, Panter‐Brick, & Stein, 2012), while levels of human insecurity—a measure of fear and worry for oneself and one’s family (Ziadni et al., 2011)—are salient in this environment, and predict mental health recovery (Panter‐Brick, Dajani, et al., 2018).

## Conceptual Model and Hypotheses

**Figure 1:**
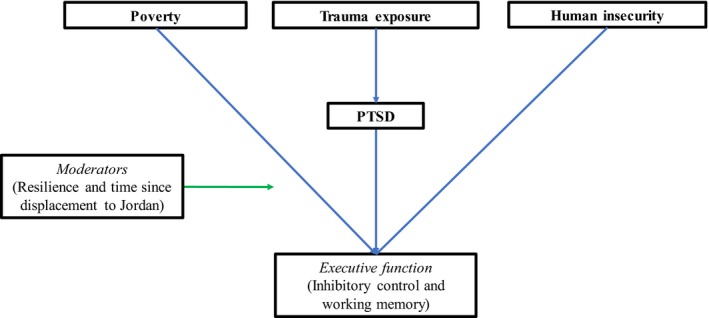
Conceptual model linking dimensions of adversity and executive function. Tested covariates include baseline task performance, refugee status, age, gender, child education, mother education, and father education. PTSD = posttraumatic stress disorder. [Color figure can be viewed at http://wileyonlinelibrary.com]

Figure 1 presents a conceptual model linking types of childhood adversity with two measures of EF. It guided our theoretical expectations as we examined the relative strength of associations with poverty, trauma exposure, PTSD, and insecurity. We hypothesized that—controlling for trauma, PTSD, and human insecurity—higher levels of poverty would be associated with weaker IC and WM task performance. Given previous work in the United States demonstrating a lack of association between trauma exposure and EF, we hypothesized that associations between trauma exposure or PTSD and EF task performance would likely be seen in Syrian refugees, but not Jordanians, as the former had higher exposures to war violence. Given research documenting the regional importance of human insecurity, we hypothesized that higher levels of human insecurity would be negatively associated with IC and WM task performance. Finally, we examined resilience and migration history as potential moderators. We first examined associations in our whole sample (*n* = 450), then for refugees (*n* = 240) and non‐refugees (*n* = 210) separately. The analysis of data collected among refugee and non‐refugee populations living side‐by‐side in Jordan provides the variation needed to disentangle the effects of different dimensions of childhood adversity on EF (Table 1).

**Table 1:** Participant Characteristics

	Syrian refugees (*n* = 240)	Jordanian non‐refugees (*n* = 210)	Both samples combined (*n* = 450)
Age in years	14.18 (1.92)	14.42 (1.61)	14.29 (1.79)
% Male	57.5	57.6	57.6
Years in school (child)	6.60 (2.23)	7.78 (1.77)	7.15 (2.11)*
Primary education or less (mother), %	35.0	8.6	22.7*
Primary education or less (father), %	30.1	11.9	21.6*
Predictors			
Household wealth, *n* household items	6.65 (2.23)	10.12 (2.07)	8.27 (2.76)*
War‐related trauma exposure, *n* events	5.81 (3.07)	0.67 (1.47)	3.37 (3.55)*
% Posttraumatic stress disorder	65.1	16.2	42.2*
Human insecurity	68.25 (20.79)	62.69 (21.55)	65.65 (21.31)*
Executive function outcomes			
Inhibitory control [IC]	0.83 (0.28)	0.84 (0.26)	0.84 (0.27)
Working memory [WM]	67.84 (25.39)	68.75 (22.49)	68.27 (23.94)

Note

Unless otherwise stated, values are means (*SD*). The measure for IC is the performance across trials, where 0 is an incorrect and 1 is a correct response on each trial. The measure for WM is the average distance deviated from the target (in pixels) across trials.

* *p*<.05 difference between refugees and non‐refugees, using an independent samples t‐test for continuous variables and a chi‐square for categorical variables.

## method

The study was designed as a partnership between Mercy Corps, an international nongovernmental organization, and researchers with expertise in health and cognitive development. Fieldwork was conducted in 2015–2016 in northern Jordan, in areas heavily affected by the Syria crisis (Mercy Corps, 2013). We contacted refugee and non‐refugee participants through a Mercy Corps registry of youth considered high risk on the basis of their mental health difficulties and poor access to basic services. All study participants were eligible for Mercy Corps humanitarian programming. Syrian refugee youth had been in Jordan for 2.97 years on average (*SD* = 0.98 years).

We surveyed participants prior to the start of Mercy Corps activities (a program called *Advancing Adolescents*; Arabic: *Nubader*; Mercy Corps, 2013). We tracked mental health and stress outcomes over a 1‐year period for a cohort of 817 participants, 12–18 years old, representing half (48%) of all youth enrolled by Mercy Corps at the time of study. We asked 58% of our participants (*n* = 474), as many youth as could be accommodated, to additionally complete tablet‐based measures of WM and IC at the first time‐point. Given some data losses due to difficult field conditions—including lack of electricity and Internet—450 youth (Syrian refugees, *n* = 240, 57.5% male; Jordanian non‐refugees, *n* = 210, 57.6% male) had usable EF data (Table 1).

Data were collected in four cities: Irbid (20.0%), Jarash (37.6%), Mafraq (21.8%), and Zarqa (20.7%), using community centers as a base for operations. Research team members were Syrian and Jordanian women with relevant degrees and ≥3 years of experience working with refugee children. They received extensive training on the Rapid Assessment of Cognitive and Emotional Regulation (RACER) tasks. Written informed consent was obtained from community leaders and primary caregivers, and verbal assent from adolescents. The study was approved by the Prime Minister’s Office of Jordan and Yale University. We conducted assessments in a private location; during a single session (~50 min), adolescents completed survey instruments, as well as tablet‐based RACER tasks (first IC, then WM), optimized for use in LMIC and humanitarian settings (Ford, Kim, Brown, Aber, & Sheridan, 2019).

For the predictor variables, we used a checklist of 12 household items to measure poverty (Panter‐Brick, Eggerman, Gonzalez, & Safdar, 2009; Panter‐Brick, Hadfield, et al., 2018); the *Traumatic Events Checklist* to measure trauma (Panter‐Brick et al., 2009); the eight‐item Child Revised Impact of Events Scale (Smith, Perrin, Yule, Hacam, & Stuvland, 2002) to measure PTSD symptoms; and the *Human Insecurity Scale*, developed in the West Bank for Palestinian youth (Hamayel, Ghandour, Abu Rmeileh & Giacaman, 2014; Ziadni et al., 2011) to measure fear, worry, and perceived insecurity. For moderators, we used the Arabic *Child and Youth Resilience Measure* to measure resilience (Panter‐Brick, Hadfield, et al., 2018; Ungar & Liebenberg, 2011) and asked Syrian caregivers when they had arrived in Jordan to measure recency of forced migration. For sociodemographic covariates, we recorded refugee status, age, gender, child education, mother education, and father education. The first three were self‐reported, while the latter three were reported by caregivers (see Appendix S1).

The tablet‐based RACER cognitive tasks took 2–4 min each. While participants had limited experience with these tablet‐based games, they were broadly familiar with computerized games (32.0% had a computer and 78.8% had a cell phone at home). To learn the rules of each game, adolescents completed practice trials until reaching 75% accuracy or practicing four times—whichever came first. We assessed IC and WM by measuring performance on “challenge trials” while controlling for performance on baseline trials. Thus, our measure of IC and WM is the decrement in performance caused by increasing the IC or WM task demands, controlling for all other aspects of baseline task performance, such as visual–spatial ability or familiarity with computerized games. Performance on baseline trials (Table S1) was not central to our investigation.

### Inhibitory Control

The RACER IC game is based on the “Simon task” (O’Leary & Barber, 1993; Simon & Rudell, 1967). On each trial of 60 trials, the adolescent was shown a dot on either the left or the right side of the screen, appearing in a random order (Figure S1a). On half the trials, the adolescent was presented with a solid dot, which participants were taught to touch as fast as possible (baseline trials). On the other half, a striped dot was presented; participants were taught to touch on the opposite side of the screen in this instance (challenge trials; Figure S1a). Dots were presented in a pseudorandom order, counterbalanced for side of screen and condition. Dots were presented for 2.5 s or as soon as the respondent touched the screen, whichever came first. Each trial was followed by a 0.5 s intertrial interval. The outcome for this task is accuracy, a variable indicating whether participants got the trial wrong or right (0 or 1 for each trial). Higher values indicate better accuracy.

### Working Memory

The RACER WM task is a variant of the widely used “spatial delayed match to sample task” (Goldman‐Rakic, 1996; Thomason et al., 2009). In each of 42 trials, participants were shown a screen that displayed one, two, or three dots for 2 s and instructed to remember where the dot(s) were located (Figure S2b). The screen then went blank (for 0.1 or 3 s), after which it changed color indicating that participants should touch the screen precisely where the dot(s) had appeared. Because remembering one item is easier than remembering multiple items, higher load trials are understood to tax WM more than lower load trials. One, two, and three load trials were presented an equal number of times, as were 0.1‐ and 3‐s delays. Conditions were fully counterbalanced. The primary outcome for the WM task is precision (log‐transformed), a continuous variable reflecting the distance between the participants’ touch and the target location on each trial. Lower values indicate better precision.

### Analysis

We examined bivariate associations between all variables of interest in the population (Table S2) and for refugees and non‐refugees (Table S3). We fit two regression models using generalized estimating equations with SPSS v.24 (IBM, Portsmouth, UK): one predicting IC and one predicting WM. Data were entered at the level of trial, with 60 trials per person for the IC models and 42 trials per person for the WM models. Cognitive trials were clustered within participants. We controlled for performance on each EF task at baseline, at the person level. For example, the measure of participants’ WM skill is their precision on the multiple‐dot trials, when controlling for their performance on the one‐dot trials (baseline). This is a strength of our analysis; each experimental test having multiple trials, we controlled for each participant’s baseline performance for EF outcomes.

We tested the effect of trial type (opposite‐ or same‐side in the IC task, number of balls in the WM task) on performance, regardless of person‐level predictors (i.e., childhood adversities, sociodemographic covariates; Table 2). We tested which covariates predicted our outcomes (IC and WM), keeping those with *p *< .05 for either IC or WM for all subsequent analyses. Baseline task performance and refugee status were included as covariates in all analyses, as were gender and child education (other covariates were not significant; Model 1, Table 3). We also tested four person‐level indicators of adversity (poverty, trauma exposure, PTSD, and human insecurity) together in a single model (Model 2, Table 3), then individually (Table S4). Based on initial associations, we tested for outcome differences by gender and PTSD (Table S5), and for interactions with resilience (Table S6) and time displaced in Jordan (Table S7).

**Table 2:** Cognitive Performance for Baseline and Challenge Trials (*n* = 450)

Task	Baseline *M* (*SD*)	Challenge *M* (*SD*)	*p*
Inhibitory control (IC)	0.96 (0.11)	0.84 (0.27)	< .001
Working memory (WM)	52.65 (31.75)	68.27 (23.94)	< .001

Note

The measure for IC is the performance across trials, where 0 is an incorrect and 1 is a correct response on each trial. The measure for WM is the average distance deviated from the target (in pixels) across trials. Differences between baseline and challenge trials were compared using paired samples *t*‐tests.

**Table 3:** Models of Associations Between Adversity and Executive Function (EF; *n* = 450)

Measure of adversity	EF					
	Inhibitory control (IC)			Working memory (WM)		
	β (*SE*)	95% CI	*p*	β (*SE*)	95% CI	*p*
Model 1						
Baseline task performance	0.12 (0.06)	0.003, 0.25,	**.045**	15.74 (1.52)	12.77, 18.72	**< .001**
Refugee status	−0.05 (0.20)	−0.44, 0.34	.813	−1.65 (1.61)	−4.80, 1.51	.307
Age	−0.02 (0.19)	−0.35, 0.38	.931	0.68 (1.21)	−1.69, 3.04	.575
Gender	0.47 (0.19)	0.08, 0.86	**.017**	−8.22 (1.58)	−11.32, −5.12	**< .001**
Child education	−0.06 (0.18)	−0.41, 0.30	.756	−3.67 (1.24)	−6.11, −1.23	**.003**
Mother education	0.37 (0.26)	−0.15, 0.88	.165	−3.18 (1.90)	−6.90, 0.54	.094
Father education	−0.06 (0.29)	−0.62, 0.50,	.827	−1.46 (2.06)	−5.36, 2.50	.477
Model 2						
Baseline task performance	0.14 (0.08)	−0.01, 0.29	.067	15.27 (1.15)	13.02, 17.53	**< .001**
Refugee status	−0.19 (0.38)	−0.93, 0.55	.614	−5.33 (2.82)	−10.86, 0.20	.059
Gender	0.57 (0.24)	0.11, 1.04	**.016**	−7.46 (1.76)	−10.90, −4.03	**< .001**
Child education	0.02 (0.18)	−0.24, 0.21	.868	−3.15 (1.04)	−5.19, −1.11	**.002**
Household wealth	−0.14 (0.16)	−0.44, 0.17	.379	−2.30 (1.13)	−4.52, −0.09	**.041**
War‐related trauma exposure	−0.09 (0.19)	−0.46, 0.27	.616	−0.94 (1.48)	−3.84, 1.95	.523
PTSD	0.07 (0.25)	−0.41, 0.56	.768	0.26 (2.19)	−4.02, 4.55	.904
Human insecurity	−0.01 (0.11)	−0.22, 0.21	.961	0.69 (0.79)	−0.86, 2.24	.384

Note

Model 1 tests which covariates predict EF in the combined sample. Model 2 controls for the significant covariates from Model 1 (refugee status, child gender, and gender education) to test the extent to which measures of adversity predict EF. For refugee status, Jordanians are the reference group. For gender, female is the reference group. For parents’ education, primary school or less is the reference group. Higher scores indicate greater household wealth. For posttraumatic stress disorder (PTSD), not having symptoms consistent with PTSD is the reference group. Models present standardized coefficients. IC has a binary logistic outcome; WM has a linear outcome (log of the distance deviated). A higher score indicates better IC; a lower score indicates better WM. Bold values indicate *p*<.05.

## Results

As expected, Syrian refugees experienced more adversity (as measured by poverty, trauma events, PTSD, and insecurity) compared to non‐refugees (Table 1). Specifically, Syrian refugees differed from Jordanian peers with respect to material wealth (6.65 vs. 10.10 household items), exposure to war‐related trauma (5.81 vs. 0.67 events), PTSD (65.0% vs. 16.2%), and human insecurity (68.25 vs. 62.91), *p *< .05 (Figure S2). While similar in age and gender breakdown, the Syrian refugees had completed fewer years in school (6.60 vs. 7.78), and their mothers (35.0% vs. 8.6%) and fathers (30.1% vs. 11.9%) were more likely to have reached only primary education, compared to the Jordanians (Table 1).

### Trial Type

Trial type impacted task performance on both tasks. Average IC performance was 12.2% less accurate on opposite‐side trials than same‐side trials (*p *< .001, Table 2). For WM, average precision on trials with multiple dots deviated on average 15.62 pixels more than trials with only one dot (*p *< .001, Table 2).

Contrary to expectations, no associations were found between IC and poverty, trauma exposure, PTSD, or human insecurity (Table 3) in the overall population. Only gender was associated with IC: Controlling for all other variables, males showed better performance than females (Table 3). By contrast, WM was associated with poverty, as well as gender and child education (Table 3). Thus, participants with greater household wealth had better performance on the WM task (β = −2.30, *p *= .041, Table 3); this effect was not moderated by levels of resilience (*p *> .05, Table S5). The association is negative, because smaller values for WM indicate less distance between touch and target, thereby better performance. Controlling for all other variables, boys and youth with more years of schooling performed better on the WM task. There were no significant associations between WM and levels of trauma exposure, PTSD, or human insecurity.

We examined these same associations separately for refugees and non‐refugees. Results for IC were the same for Syrian refugees as those for the overall sample. Poverty, male gender, and years of schooling were associated with WM (Table 4). The wealthier the household, the better the WM task performance (β = −2.38, *p* = .045), an association that was not moderated by resilience or time since forced displacement to Jordan (*p *> .05, Tables S6 and S7). There were no associations with levels of trauma exposure, PTSD, or human insecurity (Table 4). For Jordanian non‐refugees, we found no significant associations, save for boys performing better on IC and WM tasks, and participants with more years of schooling performing better on WM (Table 4).

**Table 4:** Models of Associations Between Adversity and Executive Function (EF) for Refugees (*n *= 240) and Non‐refugees (*n* = 210)

Measure of adversity	EF					
	Inhibitory control (IC)			Working memory (WM)		
	β (*SE*)	95% CI	*p*	β (*SE*)	95% CI	*p*
Syrian refugees						
Baseline task performance	0.21 (0.10)	0.02, 0.40	**.034**	16.54 (1.86)	12.90, 20.19	**< .001**
Gender	0.44 (0.33)	−0.20, 1.08	.177	−7.48 (2.44)	−12.26, −2.70	**.002**
Child education	0.02 (0.16)	−0.28, 0.32	.894	−3.03 (1.48)	−5.94, −0.12	**.041**
Household wealth	−0.12 (0.20)	−0.51, 0.26	.527	−2.38 (1.19)	−5.94, −0.12	**.045**
War‐related trauma exposure	−0.02 (0.21)	−0.43, 0.40	.934	−1.38 (1.41)	−4.15, 1.38	.327
PTSD	0.26 (0.28)	−0.29, 0.81	.355	−0.42 (2.56)	−5.44, 4.59	.869
Human insecurity	−0.12 (0.15)	0.42, 0.18	.447	1.45 (1.05)	−0.60, 3.50	.166
Jordanian non‐refugees						
Baseline task performance	0.06 (0.11)	−0.15, 0.26	.601	13.78 (1.80)	10.26, 17.31	**< .001**
Gender	0.73 (0.35)	0.04, 1.42	**.039**	−8.66 (2.57)	−13.70, −3.62	**.001**
Child education	−0.13 (0.18)	−0.48, 0.22	.470	−3.20 (1.24)	−5.64, −0.77	**.010**
Household wealth	−0.14 (0.30)	−0.73, 0.44	.631	−1.66 (1.25)	−4.12, 0.79	.185
War‐related trauma exposure	−0.30 (0.39)	−1.07, 0.47	.447	0.13 (1.79)	−3.38, 3.65	.940
PTSD	−0.21 (0.43)	−1.04, 0.63	.630	0.08 (4.41)	−8.55, 8.71	.986
Human insecurity	0.13 (0.17)	−0.30, 0.45	.447	0.01 (1.20)	−2.34, 2.36	.994

Note

For gender, female is the reference group. Higher scores indicate greater household wealth. For posttraumatic stress disorder (PTSD), not having symptoms consistent with PTSD is the reference group. Models present standardized coefficients. IC has a binary logistic outcome; WM has a linear outcome (log of the distance deviated). A higher score indicates better IC; a lower score indicates better WM. Bold values indicate *p*<.05.

## Discussion

This study is the first to disentangle the relative impact of poverty, violence, PTSD, and human insecurity on EF, for adolescents in a LMIC. It draws on measures in a population of 450 refugee and non‐refugee adolescents, sampled at the community level, in cities close to an active war zone. Syrian refugees had experienced what risk and resilience researchers consider to be “extreme adversity and stress” (Masten et al., 2012, p. 381). By contrast, Jordanian peers experienced less exposure to both poverty and the violence of war. We measured EF using computerized assessments (RACER tasks), observing task manipulation effects similar to those observed in Lebanon (Ford et al., 2019), Niger (Ford et al., 2019), and the United States (Sheridan, Kharitonova, Martin, Chatterjee & Gabrieli, 2014).

Recent work based on the United States has demonstrated a disassociation between trauma and poverty in predicting EF, whereby exposure to poverty but not traumatic violence predicts EF task performance (Lambert et al., 2016; Sheridan et al., 2017). This disassociation had never been tested in a sample with high levels of war trauma exposure and related ongoing stressors, as we did here. Consistent with such work, we did not observe associations between levels of traumatic events, PTSD, or insecurity and EF. Also, we did not observe group‐level differences between refugees and non‐refugees in EF, suggesting that war‐related trauma did not negatively impact EF.

We did find that participants in relatively poorer households performed worse on the WM task, controlling for baseline task performance, refugee status, gender, years of schooling, and other adversities (trauma exposure, PTSD, and insecurity). This finding remained significant within the most violence‐exposed group, Syrian refugees. We did not observe this association among Jordanian non‐refugees, potentially because there was not enough variance in the distribution of household wealth in this subgroup (Figure S1). We also observed that male gender and years of education were associated with better WM in both samples. These findings are consistent with other studies in LMICs showing that household material wealth, gender, and schooling can influence learning outcomes, such as language and mathematics skills, in middle childhood and early adolescence (Boyden, Dawes, & Tredoux, 2018; Obermeyer, Bott, & Sassine, 2015). However, it is striking that even with very high levels of traumatic violence exposure, we replicate distinctions between poverty and violence observed in US‐based samples (Sheridan et al., 2017). This confirms the importance of distinguishing between types of adversity exposure (McLaughlin & Sheridan, 2016).

While consistent with recent studies of U.S. children living in adversity, our finding that poverty, and not exposure to violence and trauma, worsened WM was somewhat unexpected, given the high levels of war exposure in Syrian refugees. Our study suggests that where young minds are under siege, it is the siege of ongoing poverty that is most important to skills of EF.

### Limitations

This study had many strengths, including assessments with a gender‐balanced, representative sample, using regionally validated measures of mental health and psychosocial well‐being (Panter‐Brick, Dajani, et al., 2018; Panter‐Brick, Hadfield, et al., 2018) and measures of EF sensitive to the demands of cognitive testing in under‐resourced areas (Ford et al., 2019). It also has several limitations. First, we relied on a small set of measures to assess EF. The RACER tasks do not represent the entirety of adolescents’ EF, and we did not measure other potentially important variables, such as IQ. Additional or longer tasks may have led to a more reliable estimate of each adolescent’s EF capability. Second, the RACER battery does not include normative interpretations of where EF levels would be for same‐aged adolescents: Our results should be interpreted as relative differences in WM and IC, rather than absolute measures. Third, our measure of war‐related trauma exposure was based on self‐reports of lifetime traumatic, frightening, and distressing events, data which can be biased by memory and PTSD experiences (Panter‐Brick, Grimon, Kalin, & Eggerman, 2015). Fourth, Syrian refugees in our cohort had been displaced for an average 3 years; it is possible that more nuanced measures, for more recently displaced youth, would find associations between war‐related violence and EF.

### Conclusion

Documenting the impacts of war and forced displacement on cognitive function contributes to a growing understanding of the ontology of EF skills. This in turn may guide approaches to interventions designed to help young refugees. This study is the first to identify predictors of EF skills in war‐affected adolescents using computerized task performance. It underscores the need to carefully document which dimensions of childhood adversity matter for EF. We find that the disassociation between violence and poverty in predicting EF, found in US‐based research, is also found in adolescents affected by the Syrian crisis. This would suggest that even in populations exposed to substantial violence and fear, poverty is uniquely associated with EF development.

## Supporting information


**Figure S1.** Illustration of the Rapid Assessment of Cognitive and Emotional Regulation (a) Inhibitory Control and (b) Working Memory Tasks

**Figure S2.** Distributions of Childhood Adversity Variables, for the Syrian Refugee (*n* = 240) and Jordanian Non‐Refugee (*n* = 210) Samples

**Table S1.** Measures of Childhood Adversity and Baseline Task Performance for Inhibitory Control and Working Memory (*n* = 240 Syrian Refugees, *n* = 210 Jordanian Non‐Refugees)

**Table S2.** Bivariate Correlations Between Continuous Covariates, Predictors, and Outcomes (*n* = 450)

**Table S3.** Bivariate Correlations Between Continuous Covariates, Predictors, and Outcomes (*n* = 240 Syrian Refugees, *n* = 210 Jordanian Non‐Refugees)

**Table S4.** Separate Analyses for Each Childhood Adversity, Testing Whether Each Adversity Separately Predicts Baseline Performance And executive Function

**Table S5.** Differences in Executive Function by Adolescents’ Gender and Posttraumatic Stress Disorder Status (*n* = 240 Syrian Refugees, *n* = 210 Jordanian Non‐Refugees)

**Table S6.** Testing for Interactions Between Poverty (Household Wealth) and Resilience in Predicting Working Memory

**Table S7.** Testing for an Interaction Between Poverty (Household Wealth) and Length of Time Since Being Displaced to Jordan in Predicting Working Memory in the Syrian Refugee Sample (*n* = 240)

**Appendix S1.** Method
