# Supplementary material for: Minds Under Siege: Cognitive Signatures of Poverty and Trauma in Refugee and Non‐Refugee Adolescents
Source: Child Dev. 2019 Oct 24;90(6):1856–65. doi: 10.1111/cdev.13320 (PMC6900191; doi:10.1111/cdev.13320)
Supplement: Supplementary file 1 — Figure S1. Illustration of the Rapid Assessment of Cognitive and Emotional Regulation (a) Inhibitory Control and (b) Working Memory Tasks [file CDEV-90-1856-s001.docx]

a)


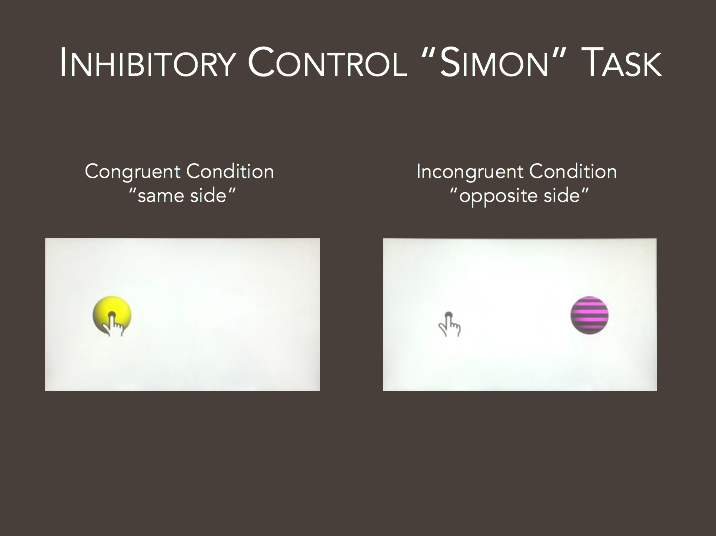


b)


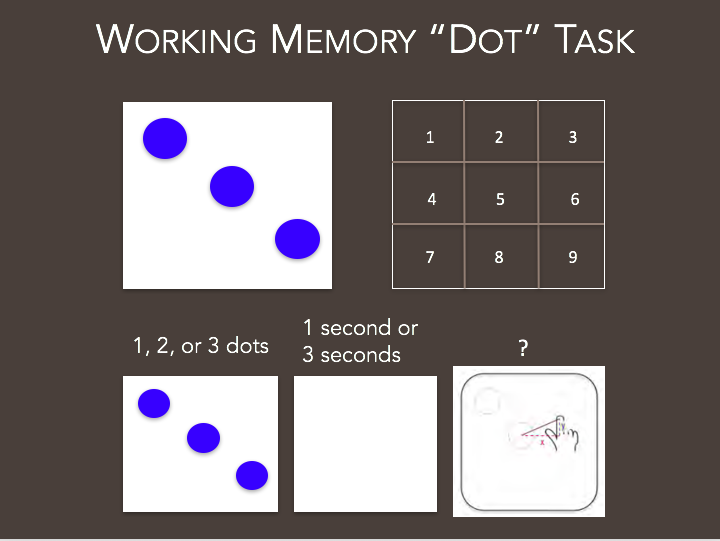


*Figure 1. Illustration of the RACER (a) inhibitory control (IC) and (b) working memory (WM) tasks. (a)* In the IC task, on every trial, participants saw a ball presented on either the right or left side of the screen. Prior to participation, children were told to press the center of the ball if it was yellow and on the opposite side of the screen if it was striped. On opposite side trials, participants required inhibitory control to suppress a prepotent response (pressing on the same side) in favor of a less usual response, pressing away from the center of the ball. Accuracy was measured as 0 or 1 on every trial indicating if they pressed on the correct side of the screen. (b) In the WM task, participants were first shown 1, 2 or 3 balls located in one of 9 pre-specified locations (see grid), upper right. Then the screen went blank for between 1 and 3 seconds. Finally, the screen changed color to grey, indicating that the participant should press on the screen where they think the dots had been located earlier. Accuracy was measured as the Euclidean distance between the press the participants made and the center of the dot which was presented previously.
