# Supplementary material for: Minds Under Siege: Cognitive Signatures of Poverty and Trauma in Refugee and Non‐Refugee Adolescents
Source: Child Dev. 2019 Oct 24;90(6):1856–65. doi: 10.1111/cdev.13320 (PMC6900191; doi:10.1111/cdev.13320)
Supplement: Supplementary file 2 — Figure S2. Distributions of Childhood Adversity Variables, for the Syrian Refugee (n = 240) and Jordanian Non‐Refugee (n = 210) Samples [file CDEV-90-1856-s002.docx]

Syrian refugee: Jordanian non-refugee:


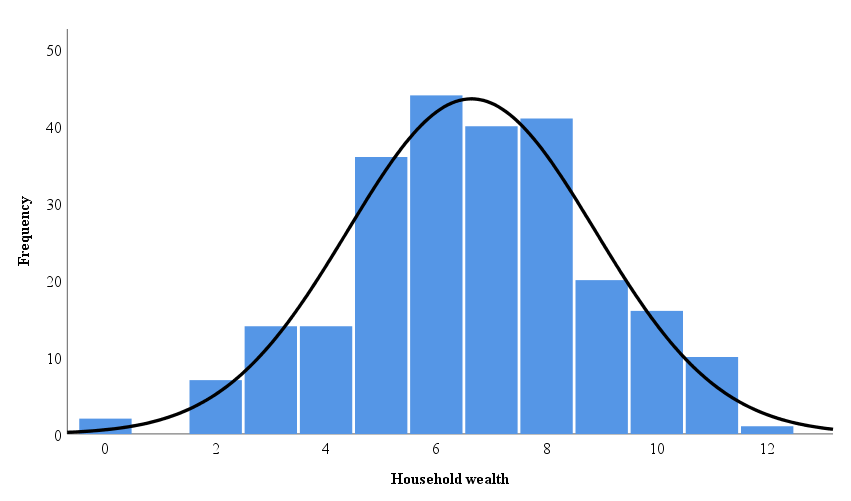

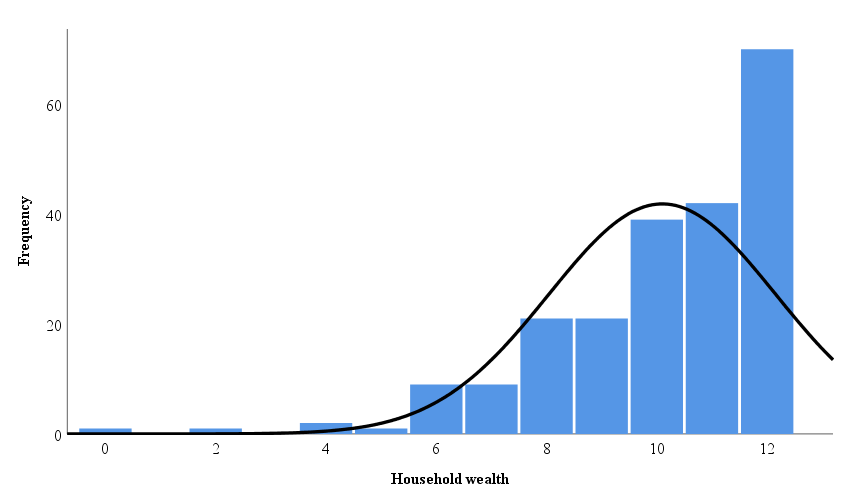


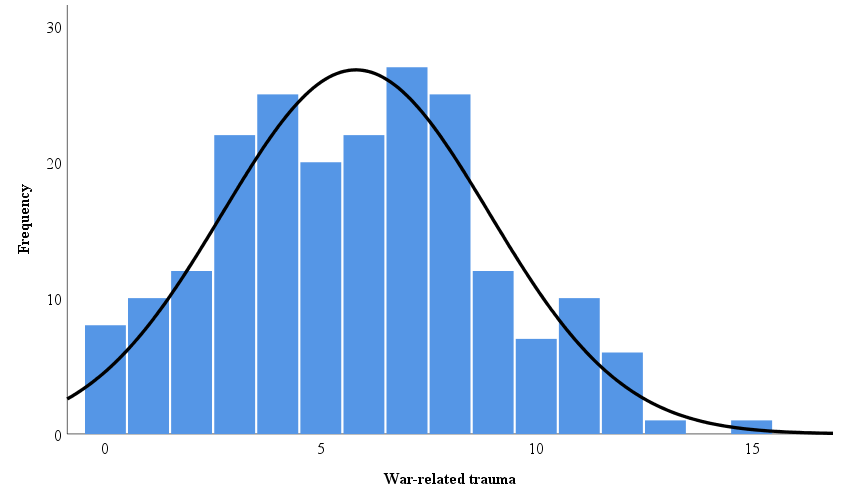

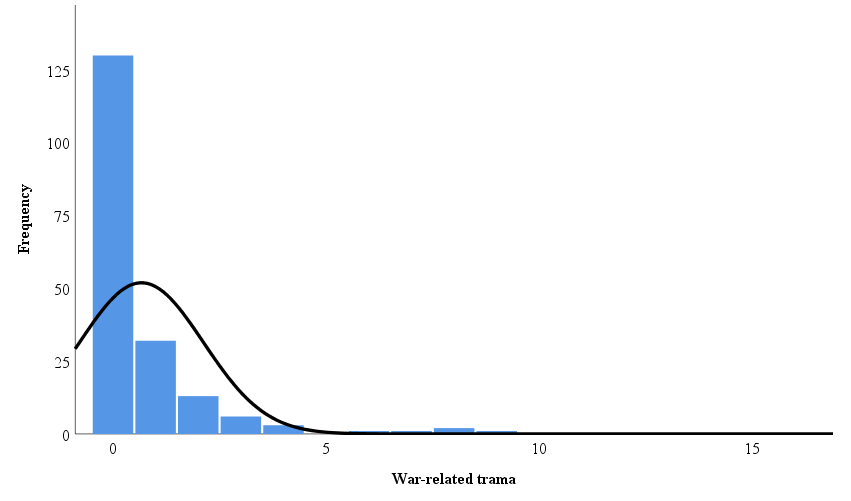


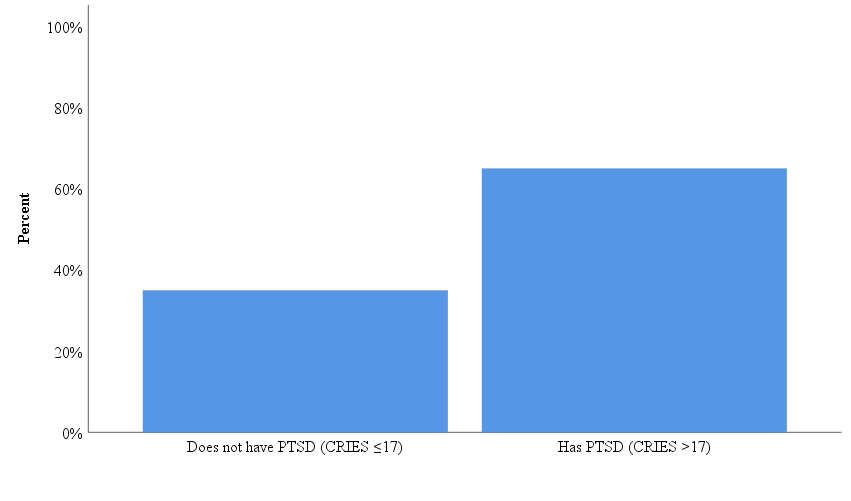

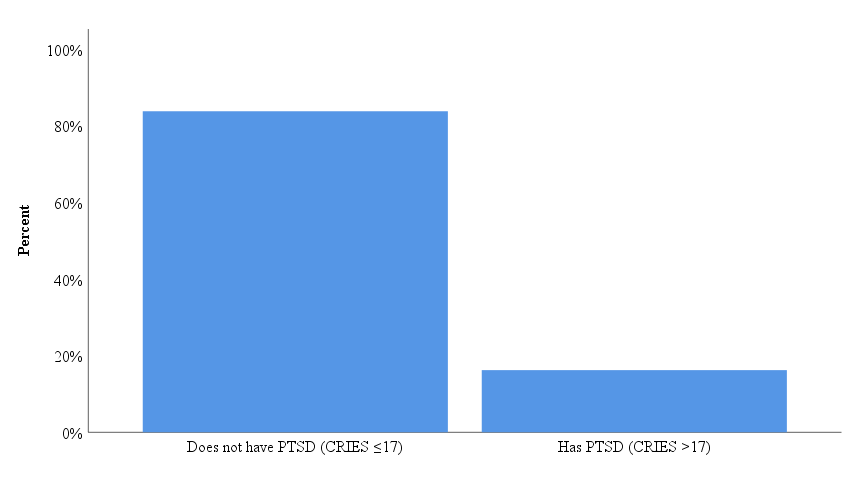


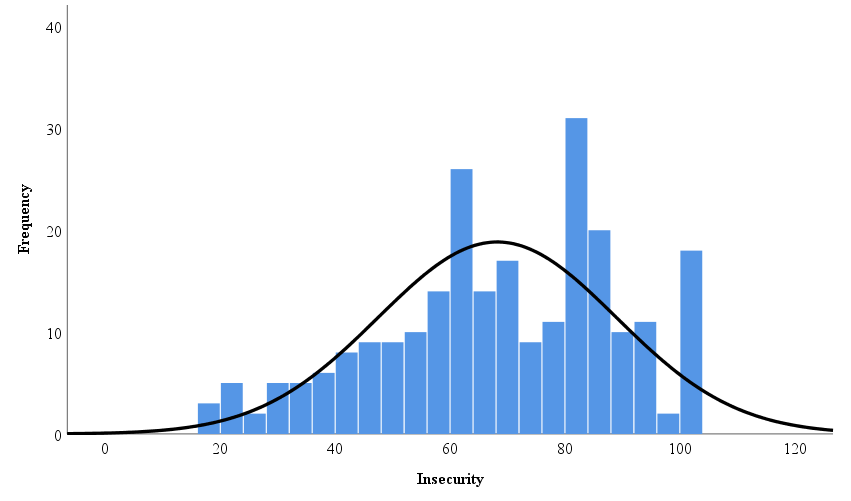

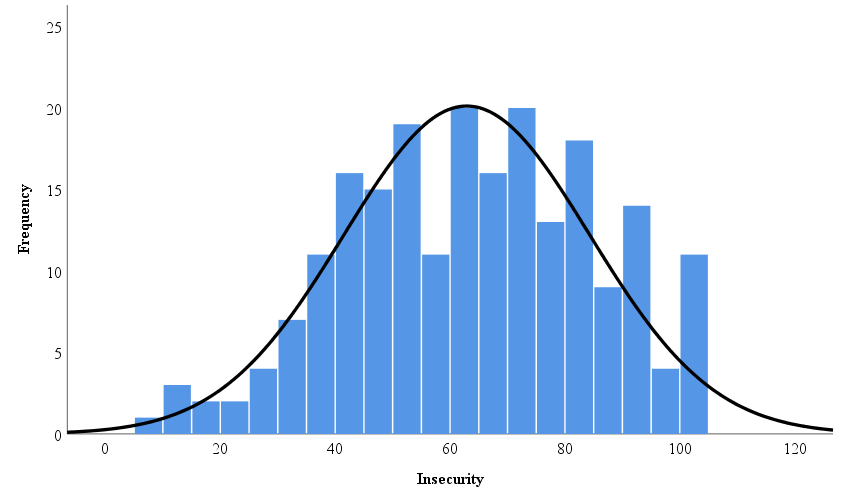


Supplemental Figure 2. *Distributions of childhood adversity variables, for the Syrian refugee (n=240) and Jordanian non-refugee (n=210) samples. PTSD = post-traumatic stress disorder, CRIES = Child Revised Impact of Events Scale.*
