# Supplementary material for: Minds Under Siege: Cognitive Signatures of Poverty and Trauma in Refugee and Non‐Refugee Adolescents
Source: Child Dev. 2019 Oct 24;90(6):1856–65. doi: 10.1111/cdev.13320 (PMC6900191; doi:10.1111/cdev.13320)
Supplement: Supplementary file 3 — Table S1. Measures of Childhood Adversity and Baseline Task Performance for Inhibitory Control and Working Memory (n = 240 Syrian Refugees, n = 210 Jordanian Non‐Refugees) [file CDEV-90-1856-s003.docx]

| Supplemental Table 1. *Measures of childhood adversity and baseline task performance for inhibitory control and working memory (n=240 Syrian refugees, n=210 Jordanian non-refugees)* | | | | | | |
| --- | --- | --- | --- | --- | --- | --- |
|  | Baseline task performance | | | | | |
|  | Inhibitory control (IC) | | | Working memory (MC) | | |
| Measure of adversity | β (SE) | 95% CI | p | β (SE) | 95% CI | p |
| Combined sample |  |  |  |  |  |  |
| Refugee status | -0.18 (0.34) | -0.85, 0.49 | .600 | 3.98 (8.11) | -11.91, 19.87 | .624 |
| Gender | -0.97 (0.27) | -1.50, -0.44 | <.001 | 1.25 (4.10) | -6.79, 9.29 | .761 |
| Child education | 0.27 (0.13) | 0.01, 0.52 | .042 | -2.87 (1.87) | -6.53, 0.80 | .125 |
| Household wealth | 0.32 (0.25) | -0.17, 0.80 | .198 | -2.15 (2.43) | -6.92, 2.61 | .376 |
| War-related trauma exposure | 0.20 (0.20) | -0.20, 0.59 | .326 | -4.38 (4.75) | -13.69, 4.93 | .356 |
| PTSD | -0.29 (0.47) | -1.22, 0.63 | .538 | -2.72 (4.13) | -10.82, 5.37 | .509 |
| Human insecurity | -0.22 (0.14) | -0.49, 0.05 | .103 | 3.79 (1.56) | 0.73, 6.85 | .015 |
| Syrian refugees |  |  |  |  |  |  |
| Gender | -0.84 (0.36) | -1.55, -0.12 | .021 | -6.91 (6.36) | -19.37, 5.55 | .277 |
| Child education | 0.21 (0.21) | -0.19, 0.62 | .297 | -5.24 (2.00) | -9.16, -1.31 | .009 |
| Household wealth | 0.16 (0.15) | -0.13, 0.44 | .280 | -6.29 (4.22) | -14.57, 1.98 | .136 |
| War-related trauma exposure | -0.03 (0.14) | -0.31, 0.25 | .846 | -5.20 (5.64) | -16.25, 5.85 | .356 |
| PTSD | -0.02 (0.41) | -0.82, 0.78 | .957 | -7.84 (6.38) | -20.34, 4.66 | .219 |
| Human insecurity | -0.43 (0.19) | -0.80, -0.07 | .021 | 6.07 (2.83) | 0.53, 11.61 | .032 |
| Jordanian non-refugees |  |  |  |  |  |  |
| Gender | -1.21 (0.38) | -1.94, -0.47 | .001 | 10.36 (4.78) | 0.99, 19.73 | .030 |
| Child education | 0.25 (0.12) | 0.02, 0.48 | .032 | 2.45 (3.39) | -4.20, 9.10 | .470 |
| Household wealth | 0.47 (0.28) | -0.08, 1.02 | .095 | 3.08 (1.94) | -0.73, 6.88 | .113 |
| War-related trauma exposure | 2.71 (1.36) | 0.05, 5.37 | .046 | 0.68 (5.56) | -10.22, 11.58 | .902 |
| PTSD | -1.25 (0.83) | -2.88, 0.38 | .131 | 2.84 (6.71) | -10.31, 15.99 | .672 |
| Human insecurity | 0.06 (0.17) | -0.28, 0.39 | .738 | 1.61 (1.64) | -1.61, 4.83 | .328 |

Models present standardized coefficients. Performance on the baseline trials of the IC ask is a binary logistic outcome; performance on the baseline trials of the WM task is a linear outcome (log of the distance deviated). A higher score indicates better performance on the baseline IC trials; a lower score indicates better performance on the baseline WM trials. Not having PTSD is the reference group. For refugee status, Jordanians are the reference group. For gender, female is the reference group. For household wealth, higher scores indicate greater relative wealth.

For the combined sample, there were no significant associations for any of our predictors except for human insecurity and WM performance: when participants reported higher insecurity, they performed worse on the baseline trials of the WM task. For Syrian refugees, high levels human insecurity was significantly associated with poorer baseline performance on both IC and WM tasks, but no significant associations were found between baseline performance on either task and our other predictors. In the non-refugee sample, war-related trauma exposure was positively associated with baseline performance on the IC task; no other associations were found for the Jordanians.
