# Supplementary material for: Minds Under Siege: Cognitive Signatures of Poverty and Trauma in Refugee and Non‐Refugee Adolescents
Source: Child Dev. 2019 Oct 24;90(6):1856–65. doi: 10.1111/cdev.13320 (PMC6900191; doi:10.1111/cdev.13320)
Supplement: Supplementary file 4 — Table S2. Bivariate Correlations Between Continuous Covariates, Predictors, and Outcomes (n = 450) [file CDEV-90-1856-s004.docx]

|  | Refugee status | Gender | Age | Child education | Resilience | Household wealth | War-related trauma exposure | PTSD | Human insecurity | Baseline task performance (IC) | Baseline task performance (WM) | Inhibitory control |
| --- | --- | --- | --- | --- | --- | --- | --- | --- | --- | --- | --- | --- |
| Gender | <.01 |  |  |  |  |  |  |  |  |  |  |  |
| Age | -.05 | -.12* |  |  |  |  |  |  |  |  |  |  |
| Child education | -.27** | -.22** | .75** |  |  |  |  |  |  |  |  |  |
| Resilience | -.19** | .08 | -.07 | .02 |  |  |  |  |  |  |  |  |
| Household wealth | -.63** | -.03 | .05 | .26** | .22** |  |  |  |  |  |  |  |
| War-related trauma exposure | .72** | .10 | .15* | -.90 | -.19** | -.53** |  |  |  |  |  |  |
| PTSD | .49** | .01 | .09* | -.08 | -.10* | -.35** | .59** |  |  |  |  |  |
| Human insecurity | .13* | -.20** | -.05 | -.05 | .15* | -.15* | .12* | .12* |  |  |  |  |
| Baseline task performance (IC) | -.02 | -.11* | .02 | .02 | .06 | .05 | -.03 | -.03 | -.06 |  |  |  |
| Baseline task performance (WM) | .02 | -.14* | -.08 | -.17* | .04 | -.13* | -.10 | -.05 | .13* | -.13* |  |  |
| Inhibitory control (IC) | -.03 | .12* | -.03 | -.04 | .06 | .01 | -.04 | .01 | -.05 | .93** | -.15* |  |
| Working memory (WM) | -.02 | -.11* | -.06 | -.15* | .05 | -.08 | -.09 | -.03 | .12* | -.12* | .95** | -.12* |

Supplemental Table 2. *Bivariate correlations between continuous covariates, predictors, and outcomes (*n*=450)*

This table includes all the continuous covariates, moderators, predictors, and outcomes, as well as a categorical predictor (posttraumatic stress disorder, PTSD) and two categorical covariates (refugee status and gender). Although length of time displaced to Jordan is also included as a moderator in analyses, it is not included here because it is only relevant to the Syrian refugee portion of the sample. For PTSD, 0=does not have symptoms consistent with PTSD, 1=has symptoms consistent with PTSD. For refugee status, 0=Jordanian non-refugee, 1=Syrian refugee. For gender, 0=female, 1=male. A higher score indicates better inhibitory control; a lower score indicates better working memory. ** *p*<.001, * *p*<.05
