# Supplementary material for: Minds Under Siege: Cognitive Signatures of Poverty and Trauma in Refugee and Non‐Refugee Adolescents
Source: Child Dev. 2019 Oct 24;90(6):1856–65. doi: 10.1111/cdev.13320 (PMC6900191; doi:10.1111/cdev.13320)
Supplement: Supplementary file 5 — Table S3. Bivariate Correlations Between Continuous Covariates, Predictors, and Outcomes (n = 240 Syrian Refugees, n = 210 Jordanian Non‐Refugees) [file CDEV-90-1856-s005.docx]

|  | Gender | Age | Child education | Resilience | Time since displacement | Household wealth | War-related trauma exposure | PTSD | Human insecurity | Baseline task performance (IC) | Baseline task performance (WM) | Inhibitory control |
| --- | --- | --- | --- | --- | --- | --- | --- | --- | --- | --- | --- | --- |
| Syrian refugees |  |  |  |  |  |  |  |  |  |  |  |  |
| Age | -.09 |  |  |  |  |  |  |  |  |  |  |  |
| Child education | -.21* | .74** |  |  |  |  |  |  |  |  |  |  |
| Resilience | .08 | -.07 | -.05 |  |  |  |  |  |  |  |  |  |
| Time since displacement | -.18* | -.07 | .02 | -.01 |  |  |  |  |  |  |  |  |
| Household wealth | -.05 | .06 | .17* | .12 | .18* |  |  |  |  |  |  |  |
| War-related trauma exposure | .16* | .35** | .18* | -.11 | -.20* | -.21* |  |  |  |  |  |  |
| Posttraumatic stress (PTSD) | -.02 | .19* | .07 | .003 | -.13* | -.13* | .37** |  |  |  |  |  |
| Human insecurity | -.20* | .02 | -.03 | .19* | -.10 | -.07 | .12 | .12 |  |  |  |  |
| Baseline task performance (IC) | -.09 | .06 | .07 | .04 | -.01 | .01 | <.01 | -.01 | -.09 |  |  |  |
| Baseline task performance (WM) | -.16* | -.06 | -.17* | .06 | -.003 | -.13 | -.22* | -.12 | .13 | -.18* |  |  |
| Inhibitory control (IC) | .10 | .02 | .01 | .04 | .01 | -.02 | .01 | .08 | -.07 | .93** | -.20* |  |
| Working memory (WM) | -.16* | -.10 | -.19* | .13 | .02 | -.14* | -.14* | -.06 | .18* | -.13* | .96** | -.13* |
| Jordanian non-refugees |  |  |  |  |  |  |  |  |  |  |  |  |
| Age | -.18* |  |  |  |  |  |  |  |  |  |  |  |
| Child education | -.26** | .81** |  |  |  |  |  |  |  |  |  |  |
| Resilience | .09 | -.11 | -.004 |  |  |  |  |  |  |  |  |  |
| Household wealth | -.01 | -.06 | .04 | .17* |  |  |  |  |  |  |  |  |
| War-related trauma exposure | .11 | .08 | .10 | -.19* |  | -.04 |  |  |  |  |  |  |
| Posttraumatic stress (PTSD) | <.01 | .04 | .05 | -.03 |  | .06 | .52** |  |  |  |  |  |
| Human insecurity | -.19* | -.11 | <.01 | .17* |  | -.11 | -.09 | <.01 |  |  |  |  |
| Baseline task performance (IC) | -.14* | -.06 | -.08 | .08 |  | .08 | -.04 | -.03 | -.02 |  |  |  |
| Baseline task performance (WM) | -.09 | -.16 | -.18* | .02 |  | -.20* | .05 | .06 | .13 | -.01 |  |  |
| Inhibitory control (IC) | .15* | -.11 | -.14* | .09 |  | .01 | -.08 | -.06 | -.02 | .93** | -.02 |  |
| Working memory (WM) | -.05 | <.01 | -.10 | -.08 |  | -.07 | .01 | .05 | .07 | -.10 | .96** | -.10 |

Supplemental Table 3. *Bivariate correlations between continuous covariates, predictors, and outcomes (*n*=240 Syrian refugees,* n*=210 Jordanian non-refugees)*

This table includes all continuous variables (covariates, moderators, predictors, and outcomes) and two categorical variables (PTSD as a predictor and gender as a covariate). Time since displacement is only applicable to the Syrian refugees. For PTSD, 0 = does not have symptoms consistent with PTSD, 1 = has symptoms consistent with PTSD. For gender, 0 = female, 1 = male. A higher score indicates better inhibitory control (IC); a lower score indicates better working memory (WM). ** p < .001, * p < .05
