# Supplementary material for: Minds Under Siege: Cognitive Signatures of Poverty and Trauma in Refugee and Non‐Refugee Adolescents
Source: Child Dev. 2019 Oct 24;90(6):1856–65. doi: 10.1111/cdev.13320 (PMC6900191; doi:10.1111/cdev.13320)
Supplement: Supplementary file 6 — Table S4. Separate Analyses for Each Childhood Adversity, Testing Whether Each Adversity Separately Predicts Baseline Performance And executive Function [file CDEV-90-1856-s006.docx]

Supplemental Table 4. *Separate analyses for each childhood adversity, testing whether each adversity separately predicts baseline performance and executive function*

|  | Inhibitory control | | | | Working memory | | | |
| --- | --- | --- | --- | --- | --- | --- | --- | --- |
|  | Model 1 | Model 2 | Model 3 | Model 4 | Model 1 | Model 2 | Model 3 | Model 4 |
|  | β (SE) | β (SE) | β (SE) | β (SE) | β (SE) | β (SE) | β (SE) | β (SE) |
| Combined sample |  |  |  |  |  |  |  |  |
| Household wealth | 0.01 (0.13) |  |  |  | -1.98 (0.80)* |  |  |  |
| War-related trauma |  | 0.10 (0.19) |  |  |  | -0.32 (0.96) |  |  |
| PTSD |  |  | -0.15 (0.24) |  |  |  | -0.22 (1.73) |  |
| Human insecurity |  |  |  | 0.04 (0.10) |  |  |  | 0.87 (0.72) |
| Syrian refugee |  |  |  |  |  |  |  |  |
| Household wealth | 0.06 (0.17) |  |  |  | -1.51 (1.11) |  |  |  |
| War-related trauma |  | 0.02 (0.21) |  |  |  | -0.66 (1.35) |  |  |
| PTSD |  |  | -0.03 (0.13) |  |  |  | 0.11 (2.16) |  |
| Human insecurity |  |  |  | 0.08 (0.14) |  |  |  | 1.23 (0.96) |
| Jordanian non-refugee |  |  |  |  |  |  |  |  |
| Household wealth | -0.03 (0.22) |  |  |  | -2.30 (1.17)* |  |  |  |
| War-related trauma |  | 0.40 (0.36) |  |  |  | 0.24 (1.40) |  |  |
| PTSD |  |  | 0.26 (0.18) |  |  |  | 0.07 (3.04) |  |
| Human insecurity |  |  |  | -0.01 (0.14) |  |  |  | 0.40 (1.09) |

In the combined sample, covariates are baseline task performance, refugee status, child gender, and child education. In either the Syrian refugee and Jordanian non-refugee sample, covariates are baseline task performance, child gender, and child education. Poverty (household wealth) is the predictor in Model 1, war-related trauma is the predictor in Model 2, PTSD status is the predictor in Model 3, and human insecurity is the predictor in Model 4. * p < .05.
