# Supplementary material for: Minds Under Siege: Cognitive Signatures of Poverty and Trauma in Refugee and Non‐Refugee Adolescents
Source: Child Dev. 2019 Oct 24;90(6):1856–65. doi: 10.1111/cdev.13320 (PMC6900191; doi:10.1111/cdev.13320)
Supplement: Supplementary file 7 — Table S5. Differences in Executive Function by Adolescents’ Gender and Posttraumatic Stress Disorder Status (n = 240 Syrian Refugees, n = 210 Jordanian Non‐Refugees) [file CDEV-90-1856-s007.docx]

Supplemental Table 5. *Differences in executive function by adolescents’ gender and PTSD status (*n*=240 Syrian refugees,* n*=210 Jordanian non-refugees*)

|  | Male | | | Female | | |
| --- | --- | --- | --- | --- | --- | --- |
|  | Has PTSD | No PTSD |  | Has PTSD | No PTSD |  |
|  |  |  |  |  |  |  |
|  | *M* (*SD*) | *M* (*SD*) | *p* | *M* (*SD*) | *M* (*SD*) | *p* |
| Combined sample | *n* = 107 | *n* = 150 |  | *n* = 82 | *n* = 109 |  |
| Inhibitory control (IC) | 0.87 (0.25) | 0.87 (0.24) | .839 | 0.81 (0.30) | 0.80 (0.30) | .813 |
| Working memory (WM) | 65.49 (20.71) | 66.46 (21.30) | .717 | 70.06 (23.21) | 72.20 (30.02) | .592 |
| Syrian refugees | *n* = 88 | *n* = 48 |  | *n* = 67 | *n* = 35 |  |
| Inhibitory control (IC) | 0.87 (0.25) | 0.84 (0.24) | .530 | 0.83 (0.28) | 0.75 (0.37) | .233 |
| Working memory (WM) | 63.99 (18.08) | 65.23 (17.96) | .704 | 70.25 (23.62) | 76.68 (44.79) | .343 |
| Jordanian non-refugees | *n* = 19 | *n* = 102 |  | *n* = 15 | *n* = 74 |  |
| Inhibitory control (IC) | 0.89 (0.24) | 0.88 (0.24) | .885 | 0.71 (0.36) | 0.82 (0.26) | .182 |
| Working memory (WM) | 72.44 (29.72) | 67.04 (22.77) | .369 | 70.07 (19.61) | 70.08 (19.61) | .877 |

Note: A higher score indicates better inhibitory control (% correct); a lower score indicates better working memory
(deviation from dot). PTSD was measured according to whether the participant scored >17 on the Child Revised Impact of Events Scale. PTSD status was missing for two male participants.
