# Supplementary material for: Minds Under Siege: Cognitive Signatures of Poverty and Trauma in Refugee and Non‐Refugee Adolescents
Source: Child Dev. 2019 Oct 24;90(6):1856–65. doi: 10.1111/cdev.13320 (PMC6900191; doi:10.1111/cdev.13320)
Supplement: Supplementary file 8 — Table S6. Testing for Interactions Between Poverty (Household Wealth) and Resilience in Predicting Working Memory [file CDEV-90-1856-s008.docx]

| Supplemental Table 6. *Testing for interactions between poverty (household wealth) and resilience in predicting working memory* | | | |
| --- | --- | --- | --- |
|  | Working memory | | |
| Measure of adversity | β (SE) | 95% CI | p |
| Combined sample |  |  |  |
| Baseline task performance | 15.78 (1.00) | 13.82, 17.74 | <.001 |
| Refugee status | -5.82 (2.86) | -11.41, -0.22 | .042 |
| Gender | -7.59 (1.79) | -11.10, -4.08 | <.001 |
| Child education | -3.25 (1.02) | -5.24, -1.25 | .001 |
| Household wealth | -2.75 (1.18) | -5.07. -0.43 | .020 |
| War-related trauma exposure | -0.77 (1.50) | -3.70, 2.17 | .609 |
| Posttraumatic stress disorder | 0.88 (2.21) | -3.44, 5.21 | .688 |
| Human insecurity | 0.65 (0.83) | -0.97, 2.27 | .433 |
| Resilience | 0.56 (1.00) | -1.39, 2.51 | .571 |
| Household wealth * Resilience | -0.64 (0.99) | -2.59, 1.31 | .519 |
| Syrian refugees |  |  |  |
| Baseline task performance | 14.60 (1.21) | 12.22, 16.98 | <.001 |
| Gender | -7.74 (2.48) | -12.60, -2.88 | .002 |
| Child education | -3.05 (1.35) | -5.69, -0.40 | .024 |
| Household wealth | -3.57 (1.45) | -6.40, -0.73 | .014 |
| War-related trauma exposure | -1.17 (1.66) | -4.43, 2.09 | .482 |
| Posttraumatic stress disorder | 0.49 (2.60) | -4.60, 5.58 | .850 |
| Human insecurity | 1.16 (1.14) | -1.07, 3.39 | .307 |
| Resilience | 2.99 (1.35) | 0.34, 5.65 | .027 |
| Household wealth * Resilience | 0.59 (1.44) | -2.24, 3.42 | .681 |

Models present standardized coefficients. Performance on the working memory task is a linear outcome (log of the distance deviated). A lower score indicates better working memory. Not having PTSD is the reference group. For refugee status, Jordanian non-refugee is the reference group. For adolescent’s gender, female is the reference group. For household wealth, higher scores indicate greater relative wealth.
