# Supplementary material for: Minds Under Siege: Cognitive Signatures of Poverty and Trauma in Refugee and Non‐Refugee Adolescents
Source: Child Dev. 2019 Oct 24;90(6):1856–65. doi: 10.1111/cdev.13320 (PMC6900191; doi:10.1111/cdev.13320)
Supplement: Supplementary file 9 — Table S7. Testing for an Interaction Between Poverty (Household Wealth) and Length of Time Since Being Displaced to Jordan in Predicting Working Memory in the Syrian Refugee Sample (n = 240) [file CDEV-90-1856-s009.docx]

| Supplemental Table 7. *Testing for an interaction between poverty (household wealth) and length of time since being displaced to Jordan in predicting working memory in the Syrian refugee sample (*n = *240*) | | | |
| --- | --- | --- | --- |
|  | Working memory | | |
| Measure of adversity | β (SE) | 95% CI | *p* |
| Baseline task performance | 13.99 (1.56) | 10.93, 17.04 | <.001 |
| Gender | -7.85 (2.42) | -12.59, -3.11 | .001 |
| Child education | -2.91 (1.40) | -5.65, -0.16 | .038 |
| Household wealth | -2.88 (1.54) | -5.89, 0.13 | .061 |
| War-related trauma exposure | -1.93 (1.68) | -5.23, 1.36 | .251 |
| Posttraumatic stress disorder | -0.14 (2.54) | -5.13, 4.84 | .955 |
| Human insecurity | 1.30 (1.08) | -0.82, 3.42 | .230 |
| Time in Jordan (in years) | -0.65 (0.97) | -2.56, 1.26 | .507 |
| Household wealth * Time in Jordan | -1.29 (1.22) | -3.67, 1.10 | .289 |

Models present standardized coefficients. Performance on the working memory task is a linear outcome (log of the distance deviated). A lower score indicates better working memory. Not having PTSD is the reference group. For adolescent’s gender, female is the reference group. For household wealth, higher scores indicate greater relative wealth.
