# Supplementary material for: Minds Under Siege: Cognitive Signatures of Poverty and Trauma in Refugee and Non‐Refugee Adolescents
Source: Child Dev. 2019 Oct 24;90(6):1856–65. doi: 10.1111/cdev.13320 (PMC6900191; doi:10.1111/cdev.13320)
Supplement: Supplementary file 10 — Appendix S1. Method [file CDEV-90-1856-s010.docx]

**Supplementary Methods**

*Poverty.* We used a checklist of household material items, a culturally-relevant and reliable way to measure relative wealth in conflict settings (Panter-Brick, Dajani, et al., 2018; Panter-Brick, Eggerman, Gonzalez, & Safdar, 2009). Parents were asked which of a list of 12 pre-specified items they had in their household: satellite dish, computer, car, functioning TV, smartphone, refrigerator, oven with gas, bedframe, washing machine, heater, fan, and water heater. Lower total scores indicate greater poverty.

*Trauma exposure.* We used the *Traumatic Events Checklist*, which has been adapted from the Harvard Trauma Questionnaire and the Gaza Trauma Checklist for use with adolescents in conflict settings (Panter-Brick et al., 2009) to assess exposure to war-related violence. The 17 items (yes/no) are relevant to situations of armed conflict, regarding whether, for example, adolescents have “directly witnessed a bombardment or rocket explosions related to war”, or “seen someone else severely beaten, shot or killed.” A higher score indicates exposure to a greater number of violent, frightening, or traumatic events.

*Posttraumatic stress reactions:* We implemented the Arabic version of the eight-item *Child Revised Impact of Events Scale* (CRIES-8, Smith, Perrin, Yule, Hacam, & Stuvland, 2002), originally developed as the Impact of Events Scale by Horowitz, Wilner, and Alvarez (1979). Asked to recall a specific trauma event, participants answer questions such as, “Do you think about it even when you don’t mean to?” and “Do pictures about it pop into your mind?” on a 4-point Likert scale. Scores above the threshold of 17 points are predictive of symptomology consistent with the DSM-V definition of PTSD (Perrin, Meiser-Stedman, & Smith, 2005). For our analyses, we used this threshold to categorize participants as either having PTSD or not (1 = has symptoms consistent with PTSD, 0 = does not have symptoms consistent with PTSD). This questionnaire has been widely used in conflict and refugee settings (e.g. Kolltveit et al., 2012; Sarkadi et al., 2018).

*Human insecurity.* We implemented the *Human Insecurity Scale*, developed in the West Bank for Palestinian youth (Hamayel, Ghandour, Abu Rmeileh & Giacaman, 2014; Ziadni et al., 2011). The scale is a 10-item, 5-point Likert scale, with scores expressed as a percentage (0-100 points), measuring levels of fear and threats to human dignity relevant to conflict-affected settings with respect to daily life necessities and personal/family safety (α = 0.76). Items include: during the last two weeks, “to what extent did you worry/fear losing your current home?” and “to what extent did you worry/fear for the safety of your family?” Both face validity and cultural relevance of this instrument are high, the scale having being developed for populations affected by armed conflict in the Middle East region.

*Moderators*

*Resilience.* We used the Arabic *Child and Youth Resilience Measure (*CYRM-12 item, 5-point scale), specifically developed for this population to measure protective resources at individual, family, and community levels (Panter-Brick, Hadfield, et al., 2018; Ungar & Liebenberg, 2011; α = 0.77).

*Recency of forced migration*. We asked both parents and children how long had lived in their current home, and for Syrians, specifically when they left Syria for Jordan. We used this information only in our analyses of the Syrian refugees, since we were interested in the recency of forced migration.
